# The burden of headache and a health-care needs assessment in the adult population of Mali: a cross-sectional population-based study

**DOI:** 10.1186/s10194-024-01811-5

**Published:** 2024-06-27

**Authors:** Youssoufa Maiga, Seybou H. Diallo, Oumar Sangho, Leon Samuel Moskatel, Fatoumata Konipo, Abdoulaye Bocoum, Salimata Diallo, Awa Coulibaly, Mariam Daou, Housseini Dolo, Modibo Sangaré, Mohamed Albakaye, Zoumana Traoré, Thomas Coulibaly, Adama Sissoko, Guida Landouré, Boubacar Guindo, Mahamoudou Ahamadou, Mahamane Drahamane Toure, Abibatou Dembele, Habib Sacko, Cheick Abdoul Kadri Sao, Diakalia Coulibaly, Salimata Dembele, Cheick Oumar Coulibaly, Mohamadou Sanogo, Sekou Boiguilé, Julien Nizard, Robert Cowan, Timothy J. Steiner, and Andreas Husøy

**Affiliations:** 1 Department of Neurology, Gabriel Touré Teaching Hospital, Bamako, Mali; 2 Faculty of Medicine, University of Technical Sciences and Technologies, Bamako, Mali; 3 Department of Neurology, Stanford University, Palo Alto, CA USA; 4 Medicine Unit, Hospital of Mali, Bamako, Mali; 5 Faculty of Medicine, University of Nantes, Nantes, France; 6 NorHEAD,Department of Neuromedicine and Movement Science, Norwegian University of Science and Technology (NTNU), Edvard Griegs gate, Trondheim, Norway; 7 Department of Neurology, University of Copenhagen, Copenhagen, Denmark; 8 Division of Brain Sciences, Imperial College London, London, UK

**Keywords:** Headache, Medication-overuse headache, Epidemiology, Burden of disease, Health policy, Mali, Sub-Saharan Africa, Global Campaign against headache

## Abstract

**Background:**

Our recent studies have shown headache disorders to be very common in the central and western sub-Saharan countries of Benin and Cameroon. Here we report headache in nearby Mali, a strife-torn country that differs topographically, culturally, politically and economically. The purposes were to estimate headache-attributed burden and need for headache care.

**Methods:**

We used cluster-random sampling in seven of Mali’s eleven regions to obtain a nationally representative sample. During unannounced household visits by trained interviewers, one randomly selected adult member (18–65 years) from each household was interviewed using the structured HARDSHIP questionnaire, with enquiries into headache in the last year and, additionally, headache yesterday (HY). Headache on ≥ 15 days/month (H15+) was diagnosed as probable medication-overuse headache (pMOH) when associated with acute medication use on ≥ 15 days/month, and as “other H15+” when not. Episodic headache (on < 15 days/month) was recorded as such and not further diagnosed. Burden was assessed as impaired participation (days lost from paid and household work, and from leisure activity). Need for headache care was defined by criteria for expectation of benefit.

**Results:**

Data collection coincided with the SARS-CoV-2 pandemic. The participating proportion was nonetheless extremely high (99.4%). The observed 1-year prevalence of any headache was 90.9%. Age- and gender-adjusted estimates were 86.3% for episodic headache, 1.4% for pMOH and 3.1% for other H15+. HY was reported by 16.8% with a mean duration of 8.7 h. Overall mean headache frequency was 3.5 days/month. Participants with pMOH lost more days from paid (8.8 days/3 months) and household work (10.3 days/3 months) than those with other H15+ (3.1 and 2.8 days/3 months) or episodic headache (1.2 and 0.9 days/3 months). At population level, 3.6–5.8% of all time was spent with headache, which led to a 3.6% decrease in all activity (impaired participation). Almost a quarter (23.4%) of Mali’s adult population need headache care.

**Conclusion:**

Headache is very common in Mali, as in its near neighbours, Benin and Cameroon, and associated with substantial losses of health and productivity. Need for headache care is high – a challenge for a low-income country – but lost productivity probably translates into lost gross domestic product.

## Background

Recent studies from the Global Campaign against Headache have shown that headache disorders are very common in Benin [1] and Cameroon [2], with prevalence estimates for migraine and tension-type headache (TTH) exceeding global averages (14–15% and 26% [3–5]). Also common are disorders characterized by headache on ≥ 15 days/month (H15+), important among which (from a public-health perspective) is medication-overuse headache (MOH).

These countries are in West and West-Central sub-Saharan Africa (SSA) respectively. Both are classified by the World Bank as lower-middle income countries [6].

Mali is an interior Western African country bordered by Algeria, Niger, Guinea, Senegal, Cote d’Ivoire and Burkina Faso [7]. It has no coastline; its northern part extends far into the Sahara desert [7, 8]; it is one of the hottest countries in the world [8]. The overwhelming majority of the population live in the southern savannah, with the greatest density along the border with Burkina Faso [7]. The population, presently near 23 million [9], is expected to double by 2035: although infant, child and maternal mortality rates are among the highest in SSA [7], the fertility rate (5.5 children per woman) is fourth highest in the world. Meanwhile, less than half the population are aged 18 years or over. Mali is a low-income country [10] with extreme poverty increasing rapidly [10–12]. The political situation has been unstable and characterized by conflicts since the military coup in 2012 [10]. The findings in Benin and Cameroon cannot be extrapolated to Mali.

The aims of this study were two-fold. First was to estimate the burdens attributed to headache in the adult population of Mali. The underlying question was: in a country so environmentally, economically and politically challenged, how much did headache contribute to population ill-health? The second, recognizing these challenges, was to assess need for headache care.

## Methods

### Ethics and approvals

The study was approved by the ethics committee of the Faculty of Medicine and Dentistry at the University of Technical Sciences and Technologies, Bamako, under the number 2020/209/CE/FMOS/FAPH. It was conducted in accordance with the Declaration of Helsinki [13]. All participants gave verbal consent to inclusion.

Data were gathered anonymously, and managed in accordance with data protection legislation.

### Study design

The study was a cross-sectional survey among adults in the general population of Mali, adopting the standardized sampling methodology of the Global Campaign against Headache [14]. Trained interviewers, visiting randomly selected households unannounced, employed a structured questionnaire during face-to-face interviews.

### Sampling

The study was conducted from January to October 2021.

Through multistage cluster sampling with random selection, we aimed to generate a representative sample of the adult general population (aged 18–65 years).

Firstly, we selected seven of the country’s eleven regions to reflect its ethnic and cultural diversities: (1) Kayes and (2) Koulikoro in the west, (3) Bamako in the southwest, (4) Sikasso in the south, (5) Mopti and (6) Tombouctou (Timbuktu) in the central region, and (7) Gao in the east.

Secondly, from these regions, we randomly selected health districts: from Bamako (Mali’s most populous city and the country’s Capital), four urban districts, two from each side of the Niger river, which divides Bamako into left and right banks; from Sikasso (the second most populous region), the urban district of Koutiala and rural district of Sélingué; from Mopti (the third most populous city), the urban district of Djenné and rural district of Badiangara; and from each of the less populated regions, a single district: Koniacary from Kayes and Maracacoungo from Koulikoro (both rural), the urban district of Tombouctou, and, for security reasons (rural areas being unsafe), the urban district of Gao.

Thirdly, from health-district official lists, interviewers randomly selected four villages or city areas in each district, then one or more blocks or circumscribed areas within each village or city area. Within each block, they systematically visited consecutive dwellings (omitting empty properties and commercial premises), unannounced in the first instance (“cold-calling”).

Fourthly, at each selected dwelling, the interviewer first identified the number of families living there (a family was defined as a group of people living together and sharing a kitchen). The head of each biologically unrelated family was asked to list all adult members (aged 18–65 years) living within that household. From this list, one person (the selected participant) was randomly selected for interview by the lottery method. Those refusing were counted, but not replaced from that household, in accordance with published guidelines [14]. If the selected participant was not present, another time was arranged for interview. If this and one further appointment were not kept, he or she was considered to be withholding consent and counted as a non-participant.

When the door to a selected dwelling was not answered at first visit, the dwelling was replaced by the next. This continued until the required number of participants was achieved in each selected village or city, and in the study overall.

We aimed for a minimum sample size of *N* = 2,000, again in accordance with guidelines [14].

### Enquiry

The eight interviewers were physicians or final-year medical students trained for the purpose, selected for their knowledge of the principal local languages of Mali.

The study employed modules from the Global Campaign’s Headache-Attributed Restriction, Disability, Social Handicap, and Impaired Participation (HARDSHIP) questionnaire [15], translated into Central African French in the version previously used in Cameroon [2] and Benin [1]. The interviewers first gathered demographic information, then enquired into headache with neutral screening questions (“Have you ever had a headache?” and “Have you had a headache during the last year?“). Participants who answered positively to both were asked further questions enquiring into frequency of headache and of acute medication use, and into attributable burden using selected modules from HARDSHIP [15]. The last included impaired participation (lost time from paid and household work and from social or leisure activities, utilizing the Headache-Attributed Lost Time [HALT] questionnaire [16]), willingness to pay [WTP] for effective headache treatment, and quality of life (QoL) using the WHOQoL-8 questionnaire [17]. These enquiries were supplemented by questions about headache on the previous day (“headache yesterday” [HY]), its characteristics (duration and intensity) and its impact on activities. Participants reporting no headache in the preceding year were asked only about their QoL, to provide normative data.

There was no diagnostic enquiry beyond establishing frequency and, in those reporting H15+, identifying probable medication-overuse headache (pMOH: H15 + associated with acute medication use on ≥ 15 days/month).

### Data entry and verification

All data were entered using an electronic platform for data capture (ONA, datafax or Redcap). At the end of each day the team coordinator assessed the day’s data for completeness, inconsistencies and wrong or missed entries. Following this review, the data were downloaded and kept secure at the University of Technical Sciences and Technologies, Bamako.

### Analysis

#### Demographics

Gender was recorded as male or female. Age was recorded as a continuous variable, then categorized for further analyses as 18–25, 26–35, 36–45, 46–55 or 56–65 years. The distributions of these variables were compared with those of the national population aged 18–65 years.

Marital status was recorded as single, married, widowed, separated or divorced, the last three analysed as a single category. Educational level was recorded and analysed as none, primary school, secondary school or college+. Household income was recorded in West African francs (XOF) in four categories (< 10,000; 10,000–20,000; 20,001–50,000; >50,000).

#### Headache

Participants were classified as having no headache (no headache in the last year), episodic headache (reported frequency < 15 days/month), pMOH or other H15+.

Headache-attributed burden was analysed overall and for each of these three types. Symptom burden was estimated in participants with HY from the symptoms associated with HY. Headache intensity was reported on a 3-point scale (1 = mild, 2 = moderate, 3 = severe), with means calculated as though these were continuous data. Time spent in the ictal state (TIS) was calculated as a product of duration of HY (assuming this to be typical for the participant) and headache frequency (in days/month), and reported as a proportion of total time (pTIS). These estimates, adjusted for frequency, were extrapolated to the whole sample.

Headache-attributed impaired participation recalled by participants over the preceding 3 months (HALT-90) was analysed according to established procedure: “nothing achieved” and “less than half achieved” were counted as entire days lost; to counterbalance, “more than half achieved” was reckoned as no loss, along with “everything achieved” [16]. There were separate enquiries for income-generating work (“worktime”), household chores and leisure/social activities. For those with HY, impaired participation yesterday was analysed in similar manner by counting “less than half achieved” as “nothing achieved” and “more than half achieved” as “everything achieved”. WTP was recorded in XOF/month (at the time of the study, USD 1.00 ~ XOF 590). QoL scores (in the range 8–40) were derived by summation of responses to the eight items (each on a scale of 1–5), higher scores signifying better QoL.

An assessment of headache-care need was carried out using criteria for expectation of benefit from care: (1) having pMOH or other H15+; (2) having episodic headache *and* either or both of (a) pTIS > 3.3% and/or (b) losing ≥ 3 work and/or household days over the preceding 3 months.

#### Statistics

In descriptive analyses, we used means with standard deviations (SDs) or standard errors of means (SEMs) and medians as appropriate.

We estimated 1-year prevalences of any headache, episodic headache, pMOH and other H15 + as percentages (%) with 95% confidence intervals (CIs). We adjusted observed values for age and gender according to their distributions in the national population aged 18–65 years [9]. Point prevalence of headache was calculated from reported HY, and predicted point prevalence from observed 1-year prevalence and mean reported headache frequency in days/month.

In the association analyses, demographic and social status variables were considered as independent variables and headache type as dependent. Unadjusted odds ratios (ORs) were calculated in bivariate analyses, and adjusted ORs (aORs) in multivariate analyses, each with 95% CIs. We evaluated associations between gender and symptom burden (headache frequency and duration of HY) and lost time using ANOVA. Intensity and impaired participation with HY were compared between genders using chi-squared tests. WTP and WHOQoL by headache type were analysed using ANOVA, and WHOQoL data were also displayed graphically.

Population-level estimates of pTIS, and of impaired participation in the preceding 3 months and yesterday, were derived by factoring in age- and gender-adjusted 1-year or 1-day prevalences as appropriate.

Significance was set at *p* < 0.05. We used Microsoft Excel to calculate adjusted prevalences and SPSS version 28 for all other analyses.

## Results

Data collection coincided with the SARS-CoV-2 pandemic.

### Description of sample

A total of 2,105 participants were included from the seven regions: Bamako 10.0%, Gao 10.0%, Kayes 9.5%, Koulikoro 10.4%, Mopti 20.2%, Sikasso 29.9%, Tombouctou 10.0%. There was a small preponderance of females (52.9%) compared to the general population aged 18–65 years (49.4%; chi-squared = 10.4, *p* = 0.001 [9]). The mean age of the sample was somewhat higher than in this population (35.9 vs. 33.6 years; *p* < 0.001 [9]).

There were only 12 refusals (participating proportion 99.4%).

### Headache

#### Lifetime and one-year prevalences

Lifetime prevalence of headache (headache ever) was very high (97.3%), with no difference between males (97.7% [95% CI: 96.5–98.5]) and females (97.0% [95.9–98.0]). Observed 1-year prevalence was also very high (90.9%), and similar between genders (males 90.7%; females 91.1%). Table 1shows the observed 1-year prevalence of each headache type, overall and by gender. H15 + was reported by 4.8% of participants, and diagnosed as pMOH in one third (1.6%). More females than males had pMOH (2.1% vs. 1.0%; aOR = 2.6; *p* = 0.04) and other H15+ (3.9% vs. 2.5%; aOR = 2.7; *p* = 0.01).

Adjustments for age and gender slightly increased the prevalence estimate for episodic headache (86.3% [84.7–87.7]) and decreased those for pMOH (1.4% [1.0–2.0]) and other H15+ (3.1% [2.4-4.0]), but made little difference to the estimate for any headache (90.8% [89.4–92.0]).

**Table 1:** Observed one-year prevalence of each headache type, overall and by gender

	Overall	Male	Female
	% [95% confidence interval]		
Any headache	90.9 [89.6–92.1]	90.7 [88.7–92.5]	91.1 [89.3–92.7]
All episodic headache	86.1 [84.5–87.5]	87.2 [84.8–89.2]	85.1 [82.9–87.1]
pMOH	1.6 [1.1–2.2]	1.0 [0.5–1.9]	2.1 [1.3–3.1]
Other H15+	3.3 [2.6–4.1]	2.5 [1.6–3.7]	3.9 [2.9–5.3]

pMOH: probable medication-overuse headache; H15+: headache on ≥ 15 days/month

#### Associations

No associations were found in bivariate analyses between any headache type and gender, educational level or household income (Table 2). Both pMOH and other H15 + increased with age and were most prevalent among those aged 46–55 years (OR = 3.6 [*p* = 0.02] and OR = 3.4 [*p* = 0.003] respectively), whereas episodic headache was significantly least prevalent in this age group (OR = 0.6; *p* = 0.02). Being widowed, separated or divorced was positively associated with pMOH (OR = 7.6; *p* = 0.009) and other H15+ (OR = 3.1; *p* = 0.03) and negatively associated with episodic headache (OR = 0.3; *p* < 0.001) (Table 2).

In multivariate analyses adjusting for all other demographic variables (Table 3), gender associations emerged with all three headache types: being female positively with pMOH (aOR = 2.6; *p* = 0.04) and other H15+ (aOR = 2.7; *p* = 0.01), and negatively with episodic headache (aOR = 0.6; *p* = 0.003). The positive association between age and H15 + remained significant (Table 3). Some associations with marital status were significant, particularly being married with migraine (aOR = 1.9; *p* = 0.003). There were variations but no clear trends with household income (Table 3).

#### Attributed burden

Table 4 shows frequency by headache type.

Mean headache frequency was 2.9 days/month overall, significantly higher in females (3.3 days/month) than males (2.6 days/month; *p* < 0.001). Frequency of episodic headache (2.4 days/month overall) was also significantly higher in females (2.7 days/month) than males (2.0 days/month; *p* < 0.001). Inevitably, frequencies were much higher for pMOH (28.9 days/month) and other H15+ (20.3 days/month), both similar between males and females.

**Table 2:** Bivariate analyses of associations between headache type and demographic variables

Variable	Episodic headache	pMOH	Other H15+
	Odds ratio [95% confidence interval]		
**Gender**			
male (*n* = 991)	reference	reference	reference
female (*n* = 1,114)	0.84 [0.7–1.1] *p* = 0.17	2.1 [1.0-4.4] *p* = 0.06	1.6 [1.0-2.6] *p* = 0.07
**Age** (years)			
18–25 (*n* = 622)	reference	reference	reference
26–35 (*n* = 549)	1.1 [0.8–1.5] *p* = 0.72	0.8 [0.2–2.7] *p* = 0.66	2.1 [1.0-4.4] *p* = 0.05
36–45 (*n* = 429)	0.9 [0.6–1.3] *p* = 0.50	1.5 [0.5–4.5] *p* = 0.52	2.0 [0.9–4.4] *p* = 0.08
46–55 (*n* = 263)	**0.6 [0.4–0.9]** **p** = **0.02**	**3.6 [1.3–10.3]** **p** = **0.02**	**3.4 [1.5–7.4]** **p** = **0.003**
56–65 (*n* = 242)	0.7 [0.5–1.1] *p* = 0.09	**3.5 [1.2–10.2]** **p** = **0.02**	1.9 [0.8–4.8] *p* = 0.17
**Marital status**			
single (*n* = 363)	reference	reference	reference
married (*n* = 1,675)	1.2 [0.9–1.7] *p* = 0.24	1.9 [0.6–6.3] *p* = 0.30	1.0 [0.5-2.0] *p* = 0.94
widowed, separated or divorced (*n* = 67)	**0.3 [0.2–0.6]** **p** **< 0.001**	**7.6 [1.7–34.9]** **p** = **0.009**	**3.1 [1.1–8.8]** **p** **= 0.03**
**Education level**			
none (*n* = 1,135)	0.7 [0.4–1.2] *p* = 0.17	2.5 [0.3–18.5] *p* = 0.38	1.1 [0.4–3.2] *p* = 0.84
primary (*n* = 522)	0.6 [0.3–1.2] *p* = 0.14	2.2 [0.3–17.5] *p* = 0.46	0.8 [0.3–2.4] *p* = 0.67
secondary (*n* = 322)	0.6 [0.3–1.2] *p* = 0.16	0.4 [0.0-6.3] *p* = 0.51	1.2 [0.4–3.7] *p* = 0.78
college+ (*n* = 126)	reference	reference	reference
**Household income** (XOF/month)			
< 10,000 (*n* = 188)	1.1 [0.7–1.8] *p* = 0.73	0.7 [0.1–6.6] *p* = 0.75	0.1 [0.0-1.1] *p* = 0.06
10,000–20,000 (*n* = 1,056)	1.3 [1.0-1.8] *p* = 0.10	2.6 [0.8–8.8] *p* = 0.12	0.8 [0.4–1.5] *p* = 0.51
20,001–50,000 (*n* = 473)	0.9 [0.6–1.3] *p* = 0.68	2.2 [0.6–8.4] *p* = 0.24	1.4 [0.7–2.7] *p* = 0.37
> 50,000 (*n* = 388)	reference	reference	reference

pMOH: probable medication-overuse headache; H15+: headache on ≥ 15 days/month; significant values are emboldened

**Table 3:** Multivariate analyses of associations between headache type and demographic variables

Variable	Episodic headache	pMOH	Other H15+
	Adjusted odds ratio [95% confidence interval]		
**Gender**			
Male	reference	reference	reference
Female	**0.6 [0.4–0.8]** **p = 0.003**	**2.6 [1.0-6.4]** **p = 0.04**	**2.7 [1.5–4.9]** **p = 0.01**
**Age** (years)			
18–25	reference	reference	Reference
26–35	0.9 [0.6–1.4] *p* = 0.71	0.8 [0.2–3.2] *p* = 0.76	**2.5 [1.1–5.6]** **p = 0.03**
36–45	0.7 [0.5–1.1] *p* = 0.12	1.7 [0.5–6.1] *p* = 0.43	**2.7 [1.1–6.8]** **p = 0.03**
46–55	**0.5 [0.3–0.9]** **p = 0.01**	**4.0 [1.2–13.6]** **p = 0.03**	**4.0 [1.6–10.3]** **p = 0.004**
56–65	0.6 [0.4–1.1] *p* = 0.09	**4.1 [1.1–15.7]** **p = 0.04**	2.4 [0.8–7.2] *p* = 0.13
**Marital status**			
Single	reference	reference	Reference
Married	**1.9 [1.2–2.9]** **p = 0.003**	0.7 [0.2–2.9] *p* = 0.61	**0.4 [0.1.0]** **p = 0.04**
widowed, separated or divorced	0.7 [0.3–1.5] *p* = 0.34	1.0 [0.1–7.4] *p* = 0.96	0.8 [0.2–3.1] *p* = 0.78
**Education level**			
None	0.6 [0.3–1.1] *p* = 0.10	1.3 [0.2–11.1] *p* = 0.79	1.5 [0.5–4.7] *p* = 0.47
Primary	0.5 [0.3-1.0] *p* = 0.07	1.6 [0.2–13.3] *p* = 0.69	1.1 [0.3–3.5] *p* = 0.92
Secondary	0.6 [0.3–1.2] *p* = 0.14	0.3 [0.0-4.6] *p* = 0.37	1.4 [0.4–4.7] *p* = 0.57
college+	reference	reference	Reference
**Household income** (XOF/month)			
< 10,000	1.5 [0.8–2.6] *p* = 0.20	0.4 [0.0-4.7] *p* = 0.49	**0.1 [0.0-0.7]** **p = 0.02**
10,000–20,000	**1.7 [1.2–2.6]** **p = 0.006**	1.8 [0.5–6.8] *p* = 0.41	0.6 [0.3–1.2] *p* = 0.13
20,001–50,000	1.0 [0.7–1.5] *p* = 0.98	1.9 [0.5–7.6] *p* = 0.33	1.3 [0.6–2.6] *p* = 0.53
> 50,000	reference	reference	Reference

pMOH: probable medication-overuse headache; H15+: headache on ≥ 15 days/month; significant values are emboldened

**Table 4:** Symptom burden and impaired participation attributed to headache at individual level, overall and by gender

	Overall	Male	Female	Male vs. female
**Frequency** (days/month)				
Any headache	3.5±0.1; 2.0	2.8±0.2; 1.0	4.1±0.2; 2.0	**F(1, 1793) = 25.0;***p* < 0.001
pMOH	28.9±0.8; 30.4	27.0±2.3; 30.4	29.7±0.7; 30.4	F(1, 29) = 2.0; *p* = 0.17
Other H15+	20.3±1.3; 20.0	20.9±2.0; 20.0	20.0±1.6; 20.0	F(1, 65) = 0.1; *p* = 0.72
Episodic headache	2.4±0.1; 2.0	2.0±0.1; 1.0	2.7±0.1; 2.0	**F(1, 1780) = 38.6;***p* < 0.001
	**Days lost in preceding 3 months** (mean±SEM; median)			
**HALT 1 + 2**				
Any headache	1.4±0.1; 0.0	1.1±0.1; 0.0	1.7±0.2; 0.0	**F(1, 1912) = 11.3;***p* < 0.001
pMOH	8.8±2.7; 4.0	5.2±1.8; 3.5	10.3±3.8; 5.0	F(1, 31) = 0.8; *p* = 0.39
Other H15+	3.1±0.7; 0.0	2.3±1.3; 0.0	3.6±1.1; 1.0	F(1, 67) = 0.7; *p* = 0.40
Episodic headache	1.2±0.1; 0.0	1.0±0.1; 0.0	1.5±0.1; 0.0	**F(1, 1810) = 7.1;***p* = 0.008
**HALT 3 + 4**				
Any headache	1.1±0.1; 0.0	0.4±0.1; 0.0	1.7±0.2; 0.0	**F(1, 1912) = 38.8;***p* < 0.001
pMOH	10.3±3.1; 3.0	2.5±1.1; 1.0	13.7±4.3; 4.0	F(1, 31) = 2.9; *p* = 0.10
Other H15+	2.8±0.9; 0.0	0.5±0.3; 0.0	4.0±1.3; 1.0	**F(1, 67) = 6.0;***p* = 0.02
Episodic headache	0.9±0.1; 0.0	0.4±0.1; 0.0	1.3±0.1; 0.0	**F(1, 1810) = 29.1;***p* < 0.001
**HALT 5**				
Any headache	0.3±0.0; 0.0	0.2±0.0; 0.0	0.4±0.1; 0.0	**F(1, 1939) = 11.0;***p* < 0.001
pMOH	3.7±1.4; 0.0	1.5±0.9; 0.0	4.7±1.9; 0.0	F(1, 31) = 1.1; *p* = 0.30
Other H15+	0.4±0.2; 0.0	0.1±0.1; 0.0	0.6±0.2; 0.0	F(1, 67) = 2.2; *p* = 0.14
Episodic headache	0.2±0.0; 0.0	0.2±0.0; 0.0	0.3±0.0; 0.0	**F(1, 1810) = 7.6;***p* = 0.006

pMOH: probable medication-overuse headache; H15+: headache on ≥ 15 days/month; HALT: headache-attributed lost time: questions 1 and 2 relate to work time, 3 and 4 to household chores (see text), and 5 to social or leisure activity; significant values are emboldened

**Table 5:** Symptom burden of headache yesterday and impaired participation yesterday at individual level, overall and by gender

Overall	Male	Female	Male vs. female
**Duration** (hours) (mean±SEM; median)			
8.7±0.6, 4.0	7.9± + 0.9; 3.0	9.2±0.7; 5.0	F(1, 273) = 1.3; *p* = 0.26
**Intensity** (n)			
1 (mild): 11	6	5	*X*^*2*^(2, *N* = 353) = 1.1; *p* = 0.58
2 (moderate): 465	284	181	
3 (severe): 102	63	39	
mean*	2.2	2.2	
**What done yesterday** (n)			
Everything: 178	76	102	*X*^*2*^(3, *N* = 353) = 7.0; *p* = 0.07
More than half: 95	27	68	
Less than half: 30	8	22	
Nothing: 50	17	33	
**What done yesterday** (dichotomized) (n)			
Everything: 273	103	170	*X*^*2*^(1, *N* = 353) = 1.1; *p* = 0.18
Nothing: 80	25	55	

*Treating the numerical ratings as though continuous data

Table 4 also shows impaired participation (lost time from paid work [HALT 1 + 2], household chores [HALT 3 + 4] and social or leisure activity [HALT 5]).

For headache overall, mean estimated lost time from paid work was 1.4 days/3 months. For episodic headache, mean lost work days were 1.2, or 1.8% assuming a 5-day working week, significantly higher among females (1.5 days) than males (1.0 days; *p* = 0.008) (Table 4). Much higher losses arose from pMOH: 8.8 days/3 months, reportedly higher among females (10.3 days) than males (5.2 days) although the difference was not significant (*p* = 0.39). For other H15+, mean lost work days were 3.1, or 4.8% assuming a 5-day working week.

Estimated losses from household work were much lower than from paid work among males but not females (Table 4). For episodic headache, 1.1 days/3 months werelost (males 0.4 days; females 1.3 days; *p* < 0.001); for pMOH, 10.3 days (males 2.5 days; females 13.7 days; not significant [*p* = 0.10] with small numbers); for other H15+, 2.8 days (males 0.5 days; females 4.0 days; *p* = 0.02) (Table 4).

Reported losses from social or leisure activities were lower than from work (paid or household). For episodic headache, 0.2 days/3 months were lost, significantly more among females (0.3) than males (0.2; *p* = 0.006); for pMOH, 3.7 days and other H15 + 0.4 days, with no significant differences between genders (Table 4).

#### Headache yesterday

HY was reported by 16.8% (95% CI: 15.2–18.4) of all participants, by 15.3% of those with episodic headache but by 90.3% of those with pMOH (implying daily headache for most) and 59.4% of those with other H15+. Based on the observed 1-year prevalence of 90.9% and the mean recalled headache frequency of 3.5 days/month, the predicted point prevalence of any headache was 10.6%.

The mean duration of HY was 8.7±0.6 h (Table 5), not significantly different between males (7.9 h) and females (9.2 h; *p* = 0.26). Intensity (overall mean 2.2, equating to moderate [with very few participants reporting mild headache]) was also similar between the genders (*p* = 0.58). pTIS was calculated as 4.2% (the product of mean duration and mean headache frequency divided by total available time [(8.7*3.5)/(24*30)]).

With regard to impaired participation with HY (Table 5), 50.4% reported doing everything as normal, 26.9% more than half, 8.5% less than half and 14.2% nothing. There were no significant differences between males and females.

#### Quality of life and willingness to pay

**Fig. 1:**
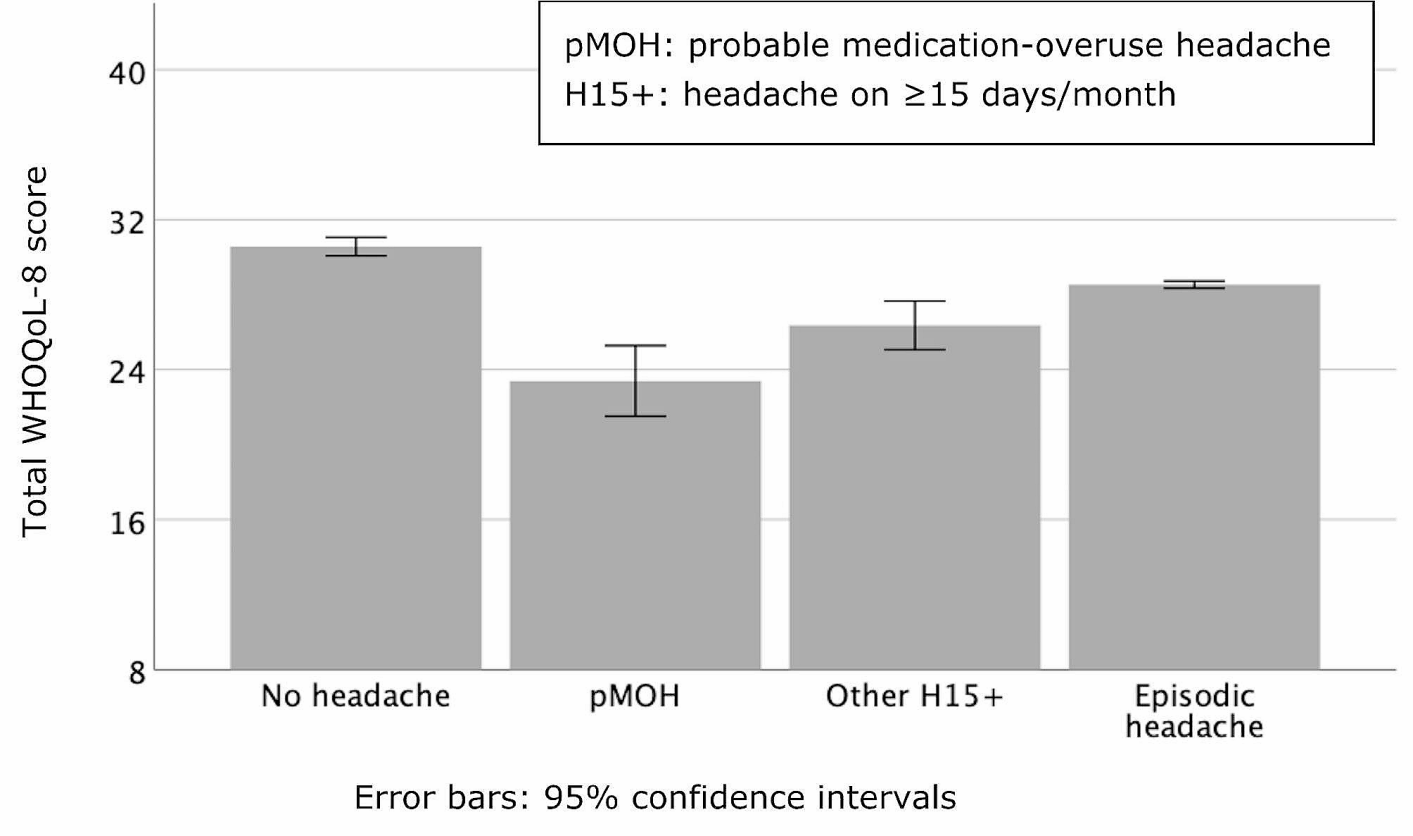
Mean reported quality of life by headache status (WHOQoL-8: possible range 8–40)

There was a significant association between headache status and QoL (*p* < 0.001; Table 6), with a clear (non-overlapping 95% CIs) declining gradient from 30.6±0.3 in those with no headache through 28.4±0.1 for episodic headache, 26.5±0.7 for other H15 + and 23.1±1.0 for pMOH (Table 6; Fig. 1).

**Table 6:** Quality of life (WHOQoL-8) and willingness to pay for effective headache treatment by headache type

**WHOQoL-8 score (range 8–40) (mean ± SEM; median)**	
Probable medication-overuse headache	23.1±1.0; 23.0
Other headache on ≥ 15 days/month	26.5±0.7; 26.0
Episodic headache	28.4±0.1; 29.0
No headache	30.6±0.3; 30.0
	**F(3, 2,101) = 38.7; ** **p < 0.001**
**Willingness to pay** (XOF/month) (mean ± SEM; median)	
Probable medication-overuse headache	9,986±2,975; 4,000
Other headache on ≥ 15 days/month	5,118±1,462; 1,000
Episodic headache	6,214±887; 1,000 F(2, 1,811) = 0.3; *p* = 0.77

Significant value is emboldened

The data on WTP were heavily skewed, as indicated by the medians. There were no significant differences between the headache types regarding WTP (Table 6). However, descriptive statistics showed that those with pMOH were willing to pay, per month, far more (XOF 9,985 [USD 16.92]) than those with other H15+ (XOF 5,118 [USD 8.67]) or episodic headache (XOF 6,214 [10.53]).

#### Headache-care needs assessment

According to our criteria, 24.7% (519/2,105) of our sample were likely to benefit from headache care (Table 7). Adjusted for age and gender, the proportion of adults judged in need of care was 23.4%: 4.5% because of H15+, the remaining 18.9% because of episodic headache.

**Table 7:** Headache-care needs assessment

Criterion fulfilled		Proportion of sample		Estimated proportion of adult population*
		**n**	%	% [95% CI]
a	Headache on ≥ 15 days/month (pMOH or other)	102	4.8	4.5 [3.7–5.5]
b	Episodic headache and pTIS > 3.3%	236	11.2	10.9 [9.6–12.3]
c	Episodic headache and ≥ 3 lost work and/or household days/3 months	343^1^	16.3	15.1 [13.6–16.7]
One or more of criteria a-c		519	24.7	23.4 [21.6–25.3]

*Age- and gender-adjusted; pMOH: probable medication-overuse headache; pTIS: proportion of time in ictal state; ^1^Of whom 81 also fulfilled criteria b

#### Population-level estimates

Table 8 shows age- and gender-adjusted population-level estimates of pTIS and impaired participation, each estimated in two ways. Based on 1-year prevalence, headache frequency and duration of HY (assuming this to be average duration for the responding participant), an estimated 3.6% of all time was spent with headache, more with episodic headache (2.3%) than with H15+ (1.4%). Based on prevalence and duration of HY, the estimate was substantially higher: 5.8% of all time.

Headache led to estimated losses, per 3 months, of 1.2 days from paid work, 0.9 days from household work and 0.3 days from social or leisure activity per person in the population (with or without headache) (Table 8). Most of this was again caused by episodic headache (1.0 workdays, 0.7 household days, 0.2 social or leisure days). According to HY data, all headache caused 3.6% of all activity to be lost.

**Table 8:** Proportion of time in ictal state and impaired participation at population level by headache type and by timeframe of enquiry (adjusted for age and gender)

Headache type	Estimated pTIS (%)		Estimated impaired participation			
	According to 1-year prevalence, average frequency, and duration of headache yesterday	According to prevalence and duration of headache yesterday	According to HALT data (lost days/3 months)			According to headache yesterday
			**Lost productivity**		**Lost social or leisure**	**Lost activity** (%)
			**Paid work**	**Household work**		
Any headache	3.6	5.8	1.2	0.9	0.3	3.6
pMOH	0.7		0.1	0.2	0.1	
Other H15+	0.7		0.1	0.1	0.0	
Episodic headache	2.3		1.0	0.7	0.2	

pTIS: proportion of time in ictal state; HALT: headache-attributed lost time; pMOH: probable medication-overuse headache; H15+: headache on ≥ 15 days/month

## Discussion

This study, diverging from standard Global Campaign methodology, avoided the difficulties associated with epidemiological diagnosis of headache type [14, 15] by looking simply at headache as a symptom associated with health and productivity losses, these being matters of interest to health and economic policy. It was not a purpose to make separate estimates for migraine and TTH, although we did for pMOH and other H15+, since the burdens associated with these highly frequent headaches were expected to be of a different order of magnitude.

However, a consequence of this approach was that we could not assess what might have been secondary headaches. While in much of the world these are relatively uncommon, malaria is endemic and prevalent in Mali, and very much associated with headache as a symptom. It is highly likely that malaria contributed to the very high reported lifetime prevalence (97.3%) and 1-year prevalence (90.8% after age- and gender-adjustment). It was less likely that it greatly influenced survey estimates based on the preceding 3 months, and very improbably those based on HY (survey participants were fit enough to be interviewed). Furthermore, during the period of data collection, the SARS-CoV-2 pandemic was happening. Headache is very much a feature of this viral disease [18]. To the extent that either of these influenced findings, they would almost certainly be reflected in H15 + rather than episodic headache. H15 + was estimated to affect one in 22 people in Mali (4.5%), with most (3.1%) not further diagnosed. The relatively few with pMOH (1.4%) were testimony, perhaps, to limited access to acute medication. However, 1.4% is within the (imprecisely) estimated global mean of 1–2% for MOH [19–21].

All this said, it is clear that in Mali, beset by all its problems, headache *is* a major factor contributing to health and productivity losses.

As for symptom burden, translating into lost health, most headache was rated of moderate intensity (assessed, with freedom from recall error) from HY. Mean duration of HY was 8.7 h. Assuming HY was typical in its duration for each participant (but, in any case, on average across the group), and with a reported mean frequency of 3.5 days/month, we estimated pTIS for those with headache at 4.2%, which diluted to 3.5% per person (with or without headache) in the general adult population. However, the pTIS estimate derived solely from HY, without the likely error associated with recall of past headache frequency, was much higher: 5.8%. The difference is obviously important. Evidenced by the discrepancy between predicted (10.6%) and observed 1-day prevalence (16.8%), it appears that people substantially underestimate headache frequency. Also possible was that participants recalled and reported attacks rather than days with headache as instructed, in which case an attack extending into the next day would be counted as one day, not two. Either way, burden measures based on reported headache frequency appear to be unreliably low.

Productivity losses were estimated at population level for all headache, both genders, at 0.9 household days per person per 3 months and at 1.2 days per person per 3 months of income-generating time, which, if this translates into gross domestic product (GDP) loss, is substantial (1.8%, assuming a 5-day working week). Total activity lost to headache, estimated from HY data, was 3.6%. In other words, this (3.6%) was the estimated proportion by which headache impaired participation in work or leisure activities throughout the adult population of Mali.

Despite their very high burdens at individual level, pMOH (mean frequency 28.9 days/month; 8.8 days/3 months lost from paid work and 10.3 days from household work) and other H15+ (20.3 days/month; 3.1 days from paid work and 2.8 days from household work), had less impact on participation and productivity at population level (factoring in prevalence) than episodic headache. At this level, more time was spent with episodic headache (2.3% of all time) than with pMOH (0.7%) and other H15+ (0.7%) combined.

QoL and WTP are regarded as all-encompassing measures, taking account of all aspects of lost wellbeing [17]. WHOQoL clearly differentiated between no headache and the various headache types, showing a gradient with pMOH, which is associated with the highest symptom burden and lost productivity, also associated with the lowest QoL. People with pMOH were, reportedly, also willing to pay most for effective treatment (almost XOF 10,000/month) than people with other headache (while this was evident in both mean and median values, the differences, with wide CIs and small numbers, were not statistically significant). In contrast WTP was almost the same for other H15+ (XOF 5,118/month) and episodic headache (XOF 6,214/month), despite QoL being lower in the former. As a measure, WTP is highly subjective, often poorly grounded in reality (HARDSHP employs the bidding-game method to address this [15]), and much influenced by ability to pay. It is worth noting that 59.1% of those who responded to the enquiry reported a monthly household income of ≤ XOF 20,000 (Table 1.

Extremely high lifetime prevalences of headache were also found in previous Global Campaign studies in Benin in western SSA (95.2% [1]) and Cameroon in central SSA (94.8% [2]), with speculation in both cases, as here, that malaria was a likely contributor. From that perspective, the three studies were in alignment. In the present study, age- and gender- adjusted 1-year prevalence of headache was also very high (90.8%), far greater than in Benin (74.9% [1]) or Cameroon (77.1% [2]). If malaria also contributed to this, it should be noted that the study predated surveillance procedures instituted in Mali from 2021 onwards [22]. But, as noted, SARS-CoV-2 may also have contributed to the observed 4.8% with H15+.

Almost one quarter of the adult population of Mali (24.7%) were judged to be in need of (likely to benefit from) headache care. Obviously this reflects the high prevalence, but the estimate was driven by attributed burden. Admittedly this estimate was derived by applying what might be considered arbitrary criteria. However, the proposition that all those with H15 + need health care is, we believe, uncontroversial. The two criteria pertaining to episodic headache (pTIS > 3.3% *or* ≥ 3 work and/or household days lost in 3 months) may be more questionable, but they are indicative of quite substantial lost health or productivity. The latter is likely to be with commensurate financial cost, making a very strong economic argument for investment in headache care, with the expectation of regaining at least some of this cost [23, 24].

### Strengths and limitations

This cross-sectional study was performed in an adequately sized, representative sample of the general population drawn randomly from eight of Mali’s eleven regions, and used standardised engagement and enquiry methodology [14]. The participating proportion was very high. These were strengths.

The principal limitation was that episodic headaches were not further diagnosed, but this did not hinder the study purposes: to estimate the burdens attributed to headache in the adult population of Mali and to assess need for headache care in this low-income and politically challenged country. Other limitations were those always associated with this type of cross-sectional research and its dependence on responses (some requiring recall over months) given at a single encounter. Enquiry into HY was a means of mitigating recall error [14, 15].

## Conclusion

Headache is very common in Mali, as in its near neighbours, Benin and Cameroon, and is associated with substantial losses of health and productivity. Need for headache care is high – a challenge for a low-income country – but lost productivity probably translates into lost gross domestic product. These are important messages for health and economic policies in Mali.

## Data Availability

The original data are held at University of Technical Sciences and Technologies, Bamako, Mali, and the analytical set at Norwegian University of Science and Technology, Trondheim, Norway. When analyses are completed, anonymised data will be available on request for academic purposes, in line with the policy of the Global Campaign against Headache.

## Abbreviations

aOR: Adjusted odds ratio
CI: Confidence interval
d/m: days/month
GDP: Gross domestic product
HARDSHIP: Headache-attributed restriction, disability, social handicap and impaired participation questionnaire
MOH: Medication-overuse headache
OR: Odds ratio
QoL: Quality of life
pMOH: Probable MOH
SD: Standard deviation
SEM: Standard error of mean
SSA: Sub-Saharan Africa
TTH: Tension-type headache
USD: United states dollar
WHO: World health organization
XOF: West african franc
